# Nationwide Surveillance of Fowl Adenovirus Infection and Coinfection With Other Diseases on Slaughter Broiler in South Korea

**DOI:** 10.1155/tbed/9353432

**Published:** 2025-01-23

**Authors:** Hye-Ryoung Kim, Hye-Soon Song, Il Jang, Tuyet Ngan Thai, Hyeon-Su Kim, Yong-Kuk Kwon, Moon Her

**Affiliations:** ^1^Avian Disease Division, Animal and Plant Quarantine Agency, 177 Hyeoksin 8-ro, Gimcheon-si, Gyeongsangbuk-do 39660, Republic of Korea; ^2^Veterinary Drugs and Biologics Division, Animal and Plant Quarantine Agency, 177 Hyeoksin 8-ro, Gimcheon-si, Gyeongsangbuk-do 39660, Republic of Korea

**Keywords:** coinfection in poultry, fowl adenovirus, inclusion body hepatitis, infectious bursal disease virus, poultry productivity

## Abstract

Fowl adenovirus (FAdV) is an infectious pathogen causing economic loss to the poultry industry worldwide. Nationwide surveillance was performed to determine the prevalence and distribution of FAdV with 725 samples collected from145 broiler farms in South Korea. A total of 64 strains were identified using PCR and phylogenetic analysis based on a sequence of hexon gene; 23 of the 64 were FAdV-11/D, 19 were FAdV-5/B, and 12 were FAdV-8b/E. FAdV-1/A and FAdV-8a/E were three strains, respectively; only one strain of FAdV-2/D was detected, and there was no FAdV-4/C. FAdV was detected very frequently at 44.1% of 145 farms, but inclusion body hepatitis (IBH) diagnosed by microscopy was confirmed in 13.8%. Poultry productivity was compared between farms with a single disease or noninfection and farms with multiple infectious diseases such as colibacillosis, antigenic variant infectious bursal disease virus (IBDV) infection, infectious bronchitis, and/or IBH. Coinfection of three or more diseases, including IBH, variant IBDV infection, and infectious bronchitis, had a more deleterious effect on poultry productivity. This study provides that prevalence of various species of FAdV and distribution with other diseases and highlights the need for comprehensive measures against multiple diseases concurrently affecting the broiler in South Korea.

## 1. Introduction

Fowl adenovirus (FAdV), belong to the family Adenoviridae and genus Aviadenovirus, is divided into five species (A to E) with 12 serotypes (1 to 8a, 8b to 11) according to the Guideline of the International Committee on Taxonomy of Viruses [1].

FAdV is distributed globally, and this virus mainly causes inclusion body hepatitis (IBH), hydropericardium syndrome (HPS), and gizzard erosion (GE) in chickens, causing enormous economic losses to the poultry industry. Strains classified as FAdV-A (serotype 1) can cause GE in chicken and quail bronchitis. FAdV-B (serotype 5) is not yet to be pathogenic. FAdV-C (serotypes 4 and 10) are responsible for IBH and HPS. FAdV-D (serotypes 2, 3, 9, and 11) and FAdV-E (serotypes 6, 7, 8a, and 8b) can cause severe liver damage known as IBH. Among them, FAdV-11/D and FAdV-8b/E are known to be pathogenic [2].

IBH caused by FAdV was reported in Korea from 2008. Mainly, FAdV-4/C, FAdV-11/D, and FAdV-8b/E from clinical cases were identified, and rarely, FAdV-1/A, FAdV-3/D FAdV-9D were also reported by academic and industrial researchers [3–6]. The number of annual cases of IBH was 55–87 cases from 2012 to 2015 and decreased to 12–29 cases after 2015. Afterward, it increased again to 68 cases in 2019 and has been decreasing until recently, according to the Korean Animal Health Integrated System reported by the Animal Health Institute of Central and Local Government. However, active surveillance to accurately determine the actual situation has never been conducted.

Moreover, coinfection with other bacterial or viral diseases is considered to exacerbate of FAdV infection. Natural complications with chicken infectious anemia virus, virulent infectious bursal disease virus (IBDV), and reovirus and experimental coinfection of variant IBDV infection may be related with or showed enhanced clinical symptoms, higher mortality, and mass economic. Avian infectious bronchitis virus (IBV) was frequently coinfected with all FAdV species [4, 7–10].

The purpose of this study is to provide the prevalence and distribution of FAdV infection in broilers through national surveillance of Korea in 2021 and to investigate coinfections with other infectious agents for understanding poultry productivity with FAdV infection.

## 2. Materials and Method

### 2.1. Samples

From April to August 2021, 145 broiler farms were randomly selected, taking samples from five dead chickens per farm at slaughterhouses with ages ranging from 24-day-old to 38-day-old that originated from each farm in nine provinces. The number of farms for surveillance at slaughterhouses was determined based on the proportions of the number of chickens slaughtered per abattoir in 2019 [11]. The dead chicken carcasses were necropsied the same day or the following day after refrigeration to avoid misinterpretation of expected postmortem changes.

During the necropsy, samples for bacteriological culture, virus detection, and histopathological examination were collected. The surface of the livers was seared with a hot spatula, and samples were obtained using a sterile swab for bacterial examination. Swab samples were subsequently cultured on blood agar and MacConkey agar plates. After incubation overnight at 37°C, a single colony was selected, and species identity was determined using the Vitek 2 system (bioMérieux, France) according to the manufacturer's instructions. Tracheas, livers, cecal tonsils, bursas, and kidneys were obtained from five carcasses per farm and were pooled by organs.

The collected organs for the virus detection were homogenized in 10% phosphate-buffered saline and stored at −70°C until processing. Sections of the liver organs with lesions, including tracheas, liver, kidneys, and bursa, were collected and fixed in 5% buffered formalin. The samples were embedded in paraffin blocks, and paraffin wax sections were cut (5 µm), dewaxed, stained with hematoxylin and eosin, and examined using light microscopy.

### 2.2. Detection of FAdV, IBDV, and IBV

DNA/RNA was extracted from homogenized samples (liver and cecal tonsil for FAdV, trachea, kidney, and cecal tonsil for IBV and bursa for IBDV) using a Maelstrom 4000 DNA/RNA auto-extraction machine (TAN bead, Taiwan). PCR and RT-PCR assays were conducted using the specific primer pairs for the hexon gene of the FAdV, VP2 gene of the IBDV, and 3′untranslated region of the IBV as described previously [12–14] (Table S1).

### 2.3. Isolation of IBV

Samples that were positive for IBV using RT-PCR were propagated in 10 days old specific pathogen-free embryonated chicken eggs at 37°C for 72 h. Allantoic fluids were harvested and centrifuged at 2851 *g* for 10 min. The supernatants were collected. RT-PCR amplifying the S1 region was used to confirm the genotype of IBV, as described previously [15].

### 2.4. Sequencing and Phylogenetic Analysis

Direct sequencing of the PCR product was performed using an ABI 3730xl DNA analyzer. The sequences of the isolated viruses were aligned and edited with CLC Main Workbench 7.7.2 (CLC bio, Aarhus, Denmark). The partial hexon gene of FAdV sequences, the partial VP2 gene of IBDVs, and the partial S1 gene of IBVs used in this study were deposited in the GenBank database under accession numbers (Table S2). Nucleotide (nt) and amino acid (aa) sequences were aligned and trimmed using the CLC Main workbench. The phylogenetic tree of FAdV was generated by a maximum likelihood method with 1000 bootstrap replications, and phylogenetic trees of IBDV and IBV were generated by a neighbor-joining method with 1000 bootstrap replications using MEGA 11 software [16], with related species published in the DDBJ/EMBL/GenBank database. The phylogenetic tree of IBDV was displayed with iTOL (Interactive Tree Of Life) [17]. Sequences showing similarities were found using a GenBank nucleotide BLAST search, and nt and aa identity were calculated using BioEdit software (version 7.2.5).

### 2.5. Broiler Production Index (PI)

The geographical location and the number of birds at slaughter of study farms were obtained from the Livestock Production Safety Management System. PI at the study farm, which corresponded to productivity, was assessed by following equation [18]. PI for individual farms was obtained from the companies operating the slaughterhouse. This indicator was compiled from the 95 broiler farms except for 50 farms, where the company did not know or refused to provide information. PI was defined as follows:

  $$\begin{array}{c} \text{Production index }\left(PI\right)=\frac{\text{mean weight at slaughter }\left(kg\right)\times \left(\text{number of live birds}÷\text{number of 1-day chicks}\right)}{\text{days at slaughter}\times \left(\text{feed intake}÷\text{total bodyweight}\right)}\times 100. \end{array}$$

### 2.6. Statistical Analysis

The PI between coinfected farms and single-infected or noninfected farms was analyzed using Student's *t*-test of Microsoft Excel software. The probability value (*p*) < 0.05 was considered statistically significant.

## 3. Result

### 3.1. Detection of FAdV

**Figure 1:**
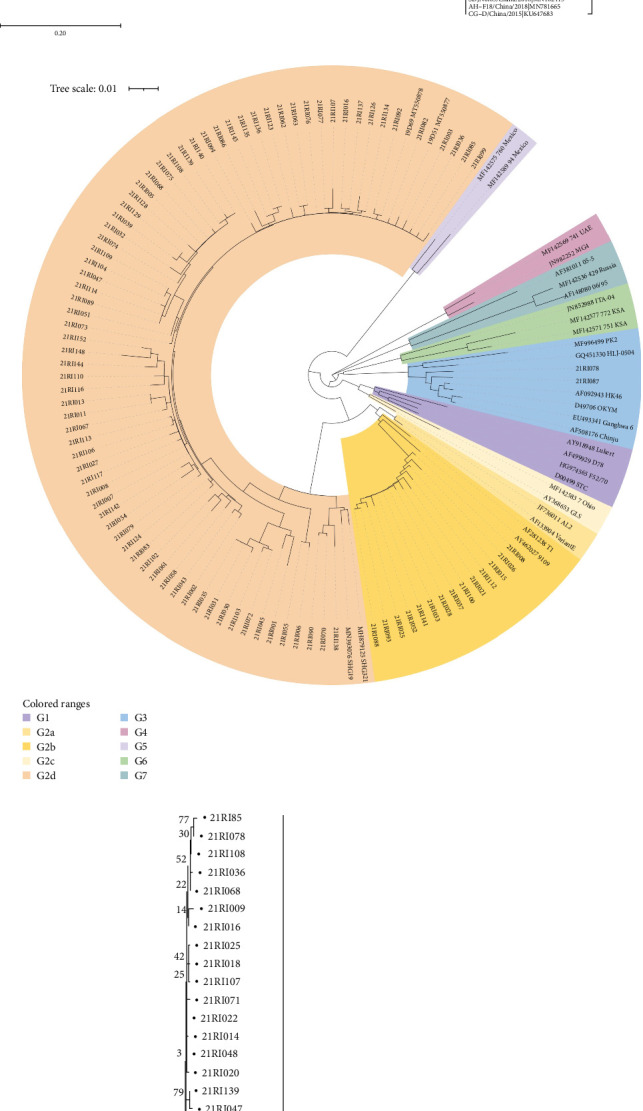
Phylogenetic tree based on the nucleotide sequences of the hexon gene of FAdV (a), VP2 gene of IBDV (b), and S1 gene of IBV (c). The trees were generated by a maximum likelihood method with 1.000 bootstrap replicates in MEGA 11 software. Green diamonds (FAdVs) and black circles (IBDVs and IBVs) indicate the newly isolated strains in this study. FAdV, fowl adenovirus; IBDV, infectious bursal disease virus; IBV, infectious bronchitis virus.

**Figure 2:**
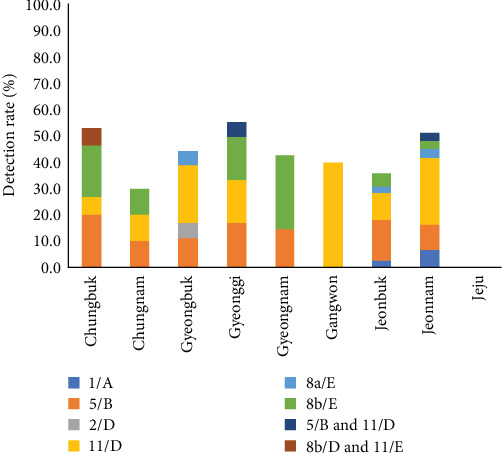
Geographical distribution of FAdV serotypes in Korea, 2021. FAdV, fowl adenovirus.

FAdV was confirmed in 64 of 145 farms, which account for 44.1% of tested farms. According to the phylogenetic analysis based on hexon gene, including the L1 loop region, FAdV-11/D was detected the most in 23 farms, FAdV-5/B was positive in 19 farms, FAdV-8b/E in 12 farms, and FAdV-8a/E and FAdV-1/A in three farms, respectively. Two species of concurrent infections were detected in three farms (two farms for FAdV-5/B and 11/D and one for FAdV-11/D and 8b/E). One farm was infected with FAdV-2/D, but FAdV-4 /C was not detected (Table 1 and Figure 1a). By region, FAdV was detected at a moderate level nationwide, with a detection rate of 30.0%–55.6% in Korea. Chungnam and Jeonbuk had relatively low detection rates compared to other regions, at 30.0% and 35.9%, respectively, and no cases were detected in Jeju (Figure 2).

### 3.2. Diagnosis of IBH

Grossly, swollen livers and petechial hemorrhages on the liver were present in a few cases infected with FAdV-8b/E and FAdV-11/D strains, but it was not a distinct lesion.

**Figure 3:**
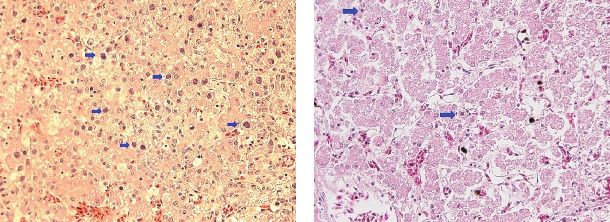
Histopathology of liver infected with FAdV-8b/E (a) and FAdV-11/E (b). Multifocal areas of necrosis and inflammation with infiltrations of mononuclear cells are present in hepatocytes of the liver. Arrows indicated basophilic intranuclear inclusion bodies. (magnification ×400). FAdV, fowl adenovirus.

Intranuclear inclusion bodies (INIB), including necrosis of hepatocytes and inflammatory cell infiltration, which are typical findings of IBH, were observed in FAdV-8b/E and FAdV-11/D infections in the histopathology. We confirmed IBH based on PCR assay for FAdV detection, sequencing, and microscopy. As a result, IBH was positive in 13.8% of the 145 farms tested. Of the 12 cases infected with FAdV-8b/E, six cases were confirmed as IBH, and of the 23 cases infected with FAdV-11/D, 12 cases were IBH. Not all cases infected with FAdV-11/D or FAdV-8b/E showed IBH (Table 1). Among cases diagnosed with IBH, microscopic lesions by FAdV-8b/E infections were evident, but in lesions caused by FAdV-11/D, INIB were rarely identified, and necrosis and infiltration inflammatory cells in the hepatocytes were weaker than lesions by FAdV-8b/E. Cases of other species of FAdV were not observed IBH (Figure 3).

### 3.3. Detection of Other Diseases

Of the 145 farms, 91 (62.8%) were confirmed to have an infection with field IBDV using RT-PCR, sequencing, and microscopic lesions. According to the phylogenetic analysis based on hypervariable regions of the VP2 gene, 75 (51.7%) farms were infected with the G2d variant, 12 (8.3%) farms were positive with the G2b variant, two farms were positive G3 virulent virus, and two farms were infected with two variant viruses together (G2b and G2d) (Figure 1b and Table 2). Histopathologically, G2 IBDV-infected bursas showed some epithelial loss, moderate lymphocyte depletion, and lymphoid follicle diffusion. G3 IBDV-infected bursas showed more severe findings. In total, 43 (29.7%) IBV-positive farms were confirmed using RT-PCR, sequencing and microscopic lesions. Three genotypes based on S1 partial gene were identified, and the predominant genotype of IBV was GI-19 (Figure 1c and Table 2). Histopathologically, IBV-infected tracheas showed loss of epithelium and lymphocyte infiltration, and GI-19 IBV-infected kidney showed damage to the renal tubular epithelial cell and lymphocyte infiltration. In total, 46 (31.7%) farms out of 145 were confirmed colibacillosis using bacterial culture and microscopic lesions.

### 3.4. Association of Productivity of Broiler and Coinfection of Other Pathogens

PI index was calculated on 95 of the farms tested. Among these, 17 farms had less (<300) productivity, and 39 farms each had good (<350) and excellent (>350) productivity. PI value criteria were referenced from a previous study [19]. Positive rates for each of the four diseases, IBH, G2 IBDV infection, IB, and colibacillosis, were lowest on farms with PI above 350, but there was no significant difference between farms with good productivity or less (< 350 and <300 of PI). The positive rates for two or more diseases were highest at 70.6% in farms with PI index of less than 300 and lowest at 46.2% in farms with PI of more than 350. This showed that coinfections with two or more diseases were significantly related to the productivity of the broiler (Table 3).

To determine whether the factor related to PI was the number of coinfection diseases or the impact of any viral disease infection, the PIs of six groups (three or more diseases coinfection, including IBH / G2 IBDV infection/IB, two diseases coinfection, including IBH / G2 IBDV Infection/IB) were analyzed. And those were compared with PI of single infected or uninfected farms. In three or more diseases coinfected farms, PI of groups, including IBH, G2 IBDV infection, and IB, showed significantly lower PI (*p* < 0.05), but there was no significant difference in PI whether or not any viral disease was included in farms with two diseases coinfection (Table 4). It means that the PI decreases significantly when infected with three or more diseases, including any one of the three diseases (IBH, IB, G2 IBDV infection).

## 4. Discussion

We performed the first active surveillance to FAdV with 725 samples collected from 145 broiler farms in South Korea, 2021. As a result of surveillance, FAdVs were detected very frequently at 64 (44.1%) out of 145 farms and evenly and moderately contaminated in eight provinces. Interestingly, FAdV-5/B, FAdV-8a/E, and FAdV-2/D were identified for the first time in Korea together with FAdV-11/D, FAdV-8b/E, and FadV-1/A associated with the symptom. Moreover, there was no FAdV-4/C species. These results seem to be different from previous reports that studied IBH and HPS clinical samples in Korea [3–5] because this was obtained through active surveillance. FAdV-5/B was detected at a high rate in boiler carcasses, but no primary disease was associated with them, similar to other reports [10, 20, 21]. FAdV-2/D and 8a/E have been reported to be associated with IBH [22], but we did not confirm IBH in this study. IBH cases were confirmed in 13.8% of 145 farms by histopathologic examination, as well as sequence-based genotyping. FAdV-11/D and FAdV-8b/E strains as etiological agents for the syndrome were identified in 15.9 % and 8.3 % out of 145 farms, but only about 50% of them caused IBH.

FAdV-4/C was not detected in this study, clearly showing that the distribution of FAdV-4/C strain is decreasing in Korea. Several inactivated vaccines to control IBH and HPS were verified in Korea. In 2015, the FAdV-4/C vaccine was approved and used in 2015, and the number of diseases caused by FAdV-4/C strains decreased significantly due to the protective effect of the vaccine [3, 6]. The relative increase of IBH caused by FAdV-11/D and FAdV-8b/E from 2017, following the decrease of IBH by FAdV-4C, highlighted the need for respective domestic vaccines [3, 6], and FAdV-8b/E vaccine was released in 2023, and the FAdV-11/D vaccine was also approved later in Korea. However, studies comparing the pathogenicity of different FAdV serotypes have shown that FAdV-4/C is more virulent than FAdV-11/D and FAdV-8b/E. FAdV-4/C, associated with IBH and HPS, causes severe clinical symptoms and high mortality rates in infected chickens, making it particularly concerning for poultry industries. In contrast, FAdV-11/D and FAdV-8b/E, while still pathogenic, generally result in milder symptoms and lower mortality rates [23–25]. Therefore, it was suspected that the damage by coinfection with other diseases was more serious than the decrease in broiler productivity caused by FAdV-8b/E and FAdV-11/D.

Coinfection with FAdV and other pathogens can significantly impact the health and immune response of poultry, often leading to higher mortality rates and more severe symptoms compared to single infections. A study on the coinfection of variant IBDV and FAdV-4/C in chickens showed increased mortality rates and reduced immune responses [7]. Coinfection with FAdV and avian reoviruses can lead to immune suppression and significant biochemical changes [9]. Synergistic effects of coinfection with multiple strains of FAdV can exacerbate disease severity [26]. The etiologic role of FAdV on broiler productivity was analyzed in coinfected farms by various etiologies in this study. As a result, it was shown that it was closely related to a decrease in productivity in farms infected with three or more diseases, including IBH, variant IBDV infection, or IB.

Under the circumstance that variant IBDV has become dominant in Korea, it seems that the impact of multiple disease infections on broiler is primarily due to the high contamination level of variant IBDV. Of the 145 farms, IBH was positive in 13.8%, but variant IBDV infection was confirmed in more than 60%, and the ratio of farms including variant IBDV infection was more than twice that of farms including IBH among farms with three or more diseases. Moreover, all farms infected with three or more diseases, including IBH, which had low productivity, were also infected with variant IBDV. The adverse impact of variant IBDV infection on poultry productivity, *E. coli* infection susceptibility, and high mortality rates with FAdV-4 infection due to immunosuppression caused by variant IBDV have been confirmed in the previous report [7, 27]. However, the seriousness of variant IBDV infection has been overlooked because it has no obvious gross lesion and is not a direct cause of death. Prompt strategies such as the commercialization of inactivated vaccines for use in breeding stocks to reduce variant IBDV infections are needed in Korea.

## 5. Conclusions

This is the first report on FAdV infection through nationwide active surveillance in Korea. FAdV-5/B, FAdV-8a/E, and FAdV-2/D were identified for the first time, and FAdV-11/D and FAdV-8b/E associated with IBH were relatively becoming dominant, but not all FAdV-11/D and FAdV-8b/E were pathogenic. The analysis of PI and coinfections revealed that broiler productivity seemed to be affected by coinfections, including variant IBDV infection and IBH. This study provides that prevalence of various species of FAdV and distribution with other diseases and highlights the need for comprehensive measures against multiple diseases concurrently affecting the broiler in South Korea.

## Figures and Tables

**Table 1:** FAdV nationwide surveillance results for 145 farms in Korea, 2021.

FAdV serotype/species	No. FAdV positive (%)	No. IBH (%)
1/A	3 (2.1)	—
5/B	19 (13.1)	—
4/C	0 (-)	—
2/D	1 (0.7)	—
11/D	23 (15.9)	12 (8.3)
8a/E	3 (2.1)	—
8b/E	12 (8.3)	6 (4.1)
5/B and 11/D	2 (1.4)	1 (<0.1)
8b/D and 11/E	1 (0.7)	1 (<0.1)

Total	64 (44.1)	20 (13.8)

Abbreviations: FAdV, fowl adenovirus; IBH, inclusion body hepatitis.

**Table 2:** IBDV and IBV nationwide surveillance results for 145 farms in Korea, 2021.

Genotypes of IBDV	No. IBDV positive (%)	Genotypes of IBV	No. IBV positive (%)
G2d (variant)	75 (51.7)	GI-19	40 (29.0)
G2b (variant)	12 (8.3)	rGI-19	2 (1.4)
G3 (virulent)	2 (1.4)	GI-15	1 (0.7)
G2b and G2d (variant mix)	2 (1.4)	—	—

Total	91 (62.8)	Total	43 (29.7)

Abbreviations: IBDV, infectious bursal disease virus; IBV, infectious bronchitis virus.

**Table 3:** Positive rates of IBH, G2 IBDV infection, IB, and colibacillosis and coinfection by PI.

PI	Number of farms	IBH (%)	G2 IBDV infection (%)	IB (%)	Colibacillosis (%)	Two or more disease coinfection (%)
<300 (less)	17	11.8	76.5	35.3	41.2	64.7
<350 (good)	39	15.4	76.9	38.5	41.0	51.3
>350 (excellent)	39	10.3	64.1	30.8	30.8	46.2

Abbreviations: IB, infectious bronchitis; IBDV, infectious bursal disease virus; IBH, inclusion body hepatitis; PI, production index.

**Table 4:** Comparison of PI between farms infected concurrently with IBH, G2 IBDV infection, IB, and colibacillosis and single infection or none.

Coinfection	Positive rate (%)	PI Mean ± SD	*p*-Value
3 or more			
Including IBH	5.3	296 ± 10.8	<0.001
Including G2 IBDV infection	14.7	314 ± 26.7	0.0057
Including IB	10.5	316 ± 30.3	0.0285
2			
Including IBH	5.3	372 ± 34.0	0.1419
Including G2 IBDV infection	34.7	337 ± 35.5	0.5140
Including IB	18.9	339 ± 33.0	0.7077
1 or noninfection	45.3	343 ± 34.6	—

*Note: p*-Value was calculated by Student's *t*-test; two samples assuming unequal or equal variances after checked by *F*-test.

Abbreviations: IB, infectious bronchitis; IBDV, infectious bursal disease virus; IBH, inclusion body hepatitis; PI, production index.

## Data Availability

The data generated or analyzed during this study are available from the corresponding author upon reasonable request.

## Nomenclature

FAdV: Fowl adenovirus
IBH: Inclusion body hepatitis
GE: Gizzard erosion
IBDV: Infectious bursal disease virus
IBV: Infectious bronchitis virus
PI: Production index
INIB: Intranuclear inclusion bodies.

## Supporting Information

Supporting Information Table S1: Primers used in this study to detect or sequence IBV, IBDV, and adenovirus. Table S2: Strains names and GenBank accession number analyzed in this study.
