# Supplementary material for: Nationwide Surveillance of Fowl Adenovirus Infection and Coinfection With Other Diseases on Slaughter Broiler in South Korea
Source: Transbound Emerg Dis. 2025 Jan 23;2025:9353432. doi: 10.1155/tbed/9353432 (PMC12016797; doi:10.1155/tbed/9353432)
Supplement: Supporting Information — Table S1: Primers used in this study to detect or sequence IBV, IBDV, and adenovirus. Table S2: Strains names and GenBank accession number analyzed in this study. [file 9353432.f1.docx]

Supplementary Table 1. Primers used in this study to detect or sequence IBV, IBDV and adenovirus.

| **Virus** | **Target gene** | **Primer sequence (5’-3’)** | **Size(bp)** | **reference** |
| --- | --- | --- | --- | --- |
| IBV | UTR | AAG GAA GAT AGG CAT GTA GCT T | 298 | [14] |
|  |  | GCT CTA ACT CTA TAC TAG CCT AT |  |  |
| IBV | S1 | AGC AAC GCC AGT TGT TAA TTT G | 750~790 | [15] |
|  |  | CWG TAC CAT TAA CAA ART AAG CMA G |  |  |
| IBDV | VP2 | GCC CAG AGT CTA CAC CAT | 579 | [13] |
|  |  | ATG GCT CCT GGG TCA AAT CG |  |  |
| adenovirus | hexon | CAA RTT CAG RCA GAC GGT | 897 | [12] |
|  |  | TAG TGA TGM CGS GAC ATC AT |  |  |

Supplementary Table 2. Strains names and GenBank accession number analyzed in this study.

| FAdV strain | GenBank  access. no | IBDV strain | GenBank  access. no. | IBV strain | GenBank  access. no. |
| --- | --- | --- | --- | --- | --- |
| 21RI102 | PQ777285 | 21RI001 | ON715027 | 21RI006 | OL688901 |
| 21RI026 | PQ777286 | 21RI002 | ON715028 | 21RI007 | OL688906 |
| 21RI121 | PQ777287 | 21RI003 | ON715029 | 21RI009 | OL688928 |
| 21RI146 | PQ777288 | 21RI005 | ON715030 | 21RI014 | OL688888 |
| 21RI011 | PQ777289 | 21RI006 | ON715031 | 21RI016 | OL688914 |
| 21RI115 | PQ777290 | 21RI007 | ON715032 | 21RI018 | OL688883 |
| 21RI002 | PQ777291 | 21RI008-G2b | ON715033 | 21RI020 | OL688899 |
| 21RI003 | PQ777292 | 21RI008-G2d | ON715034 | 21RI022 | OL688889 |
| 21RI150 | PQ777293 | 21RI011 | ON715035 | 21RI025 | OL688900 |
| 21RI027 | PQ777294 | 21RI013 | ON715036 | 21RI030 | OL688929 |
| 21RI025 | PQ777295 | 21RI015 | ON715037 | 21RI032 | OL688934 |
| 21RI138 | PQ777296 | 21RI016 | ON715038 | 21RI034 | OL688894 |
| 21RI012 | PQ777297 | 21RI021 | ON715039 | 21RI035 | OL688943 |
| 21RI017 | PQ777298 | 21RI025 | ON715040 | 21RI036 | OL688903 |
| 21RI052 | PQ777299 | 21RI026 | ON715041 | 21RI040 | OL688955 |
| 21RI062 | PQ777301 | 21RI027 | ON715042 | 21RI041 | OL688944 |
| 21RI086 | PQ777302 | 21RI028 | ON715043 | 21RI046 | OL688938 |
| 21RI135 | PQ777303 | 21RI030 | ON715044 | 21RI047 | OL688921 |
| 21RI054 | PQ777304 | 21RI031 | ON715045 | 21RI048 | OL688890 |
| 21RI051 | PQ777305 | 21RI032 | ON715046 | 21RI051 | OL688935 |
| 21RI099 | PQ777306 | 21RI033 | ON715047 | 21RI052 | OL688941 |
| 21RI029 | PQ777307 | 21RI035 | ON715048 | 21RI058 | OL688958 |
| 21RI023 | PQ777308 | 21RI036 | ON715049 | 21RI060 | OL688907 |
| 21RI066 | PQ777309 | 21RI037 | ON715050 | 21RI061 | OL691642 |
| 21RI088 | PQ777310 | 21RI039 | ON715051 | 21RI068 | OL688904 |
| 21RI044 | PQ777311 | 21RI043 | ON715052 | 21RI071 | OL688895 |
| 21RI117 | PQ777312 | 21RI045 | ON715053 | 21RI075 | OL688909 |
| 21RI053 | PQ777313 | 21RI047 | ON715054 | 21RI077 | OL688930 |
| 21RI004 | PQ777314 | 21RI051 | ON715055 | 21RI078 | OL688924 |
| 21RI106 | PQ777315 | 21RI052 | ON715056 | 21RI079 | OL688953 |
| 21RI028 | PQ777316 | 21RI054 | ON715057 | 21RI85 | OL688926 |
| 21RI092 | PQ777317 | 21RI055 | ON715058 | 21RI099 | OL688896 |
| 21RI064 | PQ777318 | 21RI058 | ON715059 | 21RI100 | OL688942 |
| 21RI116 | PQ777319 | 21RI061 | ON715060 | 21RI102 | OL688978 |
| 21RI132 | PQ777320 | 21RI062 | ON715061 | 21RI105 | OL688913 |
| 21RI001 | PQ777321 | 21RI063 | ON715062 | 21RI107 | OL688882 |
| 21RI050 | PQ777322 | 21RI067 | ON715063 | 21RI108 | OL688920 |
| 21RI010 | PQ777323 | 21RI068 | ON715064 | 21RI109 | OL688957 |
| 21RI024 | PQ777324 | 21RI070 | ON715065 | 21RI113 | OL688954 |
| 21RI045 | PQ777325 | 21RI072 | ON715066 | 21RI116 | OL688962 |
| 21RI073 | PQ777326 | 21RI073 | ON715067 | 21RI139 | OL688911 |
| 21RI074 | PQ777327 | 21RI074 | ON715068 | 21RI141 | OL688905 |
| 21RI076 | PQ777328 | 21RI075 | ON715069 | 21RI150 | OL688936 |
| 21RI144 | PQ777329 | 21RI076 | ON715070 |  |  |
| 21RI018 | PQ777330 | 21RI077 | ON715071 |  |  |
| 21RI056 | PQ777331 | 21RI078 | PQ783113 |  |  |
| 21RI109 | PQ777332 | 21RI079 | ON715072 |  |  |
| 21RI120 | PQ777333 | 21RI082 | ON715073 |  |  |
| 21RI143 | PQ777334 | 21RI083 | ON715074 |  |  |
| 21RI041 | PQ777335 | 21RI085 | ON715075 |  |  |
| 21RI055 | PQ777336 | 21RI086 | ON715076 |  |  |
| 21RI149 | PQ777337 | 21RI087 | PQ783114 |  |  |
| 21RI122 | PQ777338 | 21RI088 | ON715077 |  |  |
| 21RI119 | PQ777339 | 21RI089 | ON715078 |  |  |
| 21RI049 | PQ777340 | 21RI090 | ON715079 |  |  |
| 21RI103 | PQ777341 | 21RI092 | ON715080 |  |  |
| 21RI133 | PQ777342 | 21RI093 | ON715081 |  |  |
| 21RI040B | PQ777343 | 21RI094 | ON715082 |  |  |
| 21RI093 | PQ777344 | 21RI099 | ON715083 |  |  |
| 21RI136A | PQ777345 | 21RI100 | ON715084 |  |  |
| 21RI015B | PQ777346 | 21RI102 | ON715085 |  |  |
| 21RI111 | PQ777347 | 21RI103 | ON715086 |  |  |
| 21RI015A | PQ777348 | 21RI104 | ON715087 |  |  |
| 21RI040A | PQ777349 | 21RI106 | ON715088 |  |  |
| 21RI046 | PQ777350 | 21RI107 | ON715089 |  |  |
| 21RI136B | PQ777351 | 21RI108 | ON715090 |  |  |
| 21RI089 | PQ777352 | 21RI109 | ON715091 |  |  |
|  |  | 21RI110 | ON715092 |  |  |
|  |  | 21RI112 | ON715093 |  |  |
|  |  | 21RI113 | ON715094 |  |  |
|  |  | 21RI114 | ON715095 |  |  |
|  |  | 21RI116 | ON715096 |  |  |
|  |  | 21RI117 | ON715097 |  |  |
|  |  | 21RI123 | ON715098 |  |  |
|  |  | 21RI124 | ON715099 |  |  |
|  |  | 21RI126 | ON715100 |  |  |
|  |  | 21RI128 | ON715101 |  |  |
|  |  | 21RI129 | ON715102 |  |  |
|  |  | 21RI134 | ON715103 |  |  |
|  |  | 21RI135 | ON715104 |  |  |
|  |  | 21RI136 | ON715105 |  |  |
|  |  | 21RI137 | ON715106 |  |  |
|  |  | 21RI138 | ON715107 |  |  |
|  |  | 21RI139 | ON715108 |  |  |
|  |  | 21RI140 | ON715109 |  |  |
|  |  | 21RI141 | ON715110 |  |  |
|  |  | 21RI142 | ON715111 |  |  |
|  |  | 21RI144 | ON715112 |  |  |
|  |  | 21RI145 | ON715113 |  |  |
|  |  | 21RI148 | ON715114 |  |  |
|  |  | 21RI152 | ON715115 |  |  |
